# A Continuous Infusion Fascia Iliaca Compartment Block in Hip Fracture Patients: A Pilot Study

**DOI:** 10.4021/jocmr724w

**Published:** 2012-01-17

**Authors:** Elizabeth Dulaney-Cripe, Scott Hadaway, Ryan Bauman, Cathy Trame, Carole Smith, Becky Sillaman, Richard Laughlin

**Affiliations:** a Department of Orthopaedic Surgery, Wright State University, Dayton, OH, USA; b Department of Anesthesia, Miami Valley Hospital, Dayton, OH, USA; c Department of Orthopaedic Surgery, Miami Valley Hospital, Dayton, OH USA; d Miami Valley Hospital, Dayton, OH, USA; e Pharmacy, Miami Valley Hospital, Dayton, OH, USA

## Abstract

**Background:**

Hip fractures account for 350,000 fractures annually and the projected incidence is expected to exceed 6.3 million by 2050. As the number of hip fractures continues to increase as a result of the aging American population, the importance of limiting and preventing complications is magnified.

**Methods:**

This study demonstrated the clinical effects of a continuous fascia iliaca compartment block placed pre-operatively when combined with a comprehensive pain protocol. All patients who presented to our institution with a hip fracture were given the option of having a continuous fascia iliaca compartment block for pain control versus usual pain management (non-opioids, opioids, and ice therapy). The block was monitored by the pain service until the day of discharge from the hospital. Data was collected regarding mean pain scores, average length of stay and opioid medication use.

**Results:**

There were eighteen males and twenty four females. The pain score on post-operative day zero was reduced from a 2010 annual average of 4.1 to 1.7 in the pilot study group on the visual analog score. On post-operative day one, the 2010 annual average was 2.9 compared to 1.4 in the pilot study group. The length of stay was decreased from the 2010 annual average of 5.9 days to 4.8 days in the pilot study group. The patients used an average of 18mg of morphine equivalent medications during the average infusion time of 40.7 hours. There were no falls or infections noted within our pilot study group.

**Conclusions:**

Overall, it has been noticed that the reduction in opioid usage in this elderly patient population, with an average age of seventy five years, has produced alert and mobile patients often as early as post-operative day one. The length of stay has decreased along with the average pain score in the pilot sample of forty two patients.

**Keywords:**

Hip fracture; Fascia Iliaca Compartment Block; Pain Score

## Introduction

Hip fractures account for 350,000 fractures annually and the projected incidence is expected to exceed 6.3 million by 2050 [1]. As the number of hip fractures continues to increase as a result of the aging American population, the importance of limiting and preventing complications is magnified. The one year mortality has been documented as high as 36% [1]. Multiple strategies have been employed to reduce complications and hospital stay including pre-emptive pain medications, nerve blocks, and prompt fixation of fractures. The use of one block, a fascia iliaca compartment block has been shown to be effective in controlling pain in both hip arthroplasty and hip fracture.

Multiple studies support this finding stating that fascia iliaca compartment blocks following hip fractures are effective and easily learned [2-4]. Fascia iliaca blocks with continuous catheters have the potential to greatly reduce the morbidity in hip fracture patients when evaluating the influence and prevalence of side effects from opiate medications. Most recently, a study identified a reduction in pain by three points on the pain scale following a fascia iliaca compartment block by emergency department physicians using the two pop technique [5]. However, no large studies have examined the effect of a continuous compartment block in a hip fracture cohort. A large study, including the post-operative time period, would help validate the clinical and cost effectiveness of this low risk and highly effective compartment block. There are specific concerns regarding the elderly in the peri-operative period regarding side effects from medication. One such concern is acute delirium associated with opioid medication. Delirium has been identified as a variable that delays ambulation and necessitates placement for rehabilitation [1]. Other concerns include urinary retention and sedation. A study, though in children, identified a 34% decrease in urinary retention in the fascia iliaca compartment block in comparison to a group receiving morphine through a fascia iliaca compartment catheter following pelvic osteotomy [6].

It has been shown that patients with higher post-operative pain have an increased length of hospital stay, delayed ambulation, and long-term functional impairment [7].

Summarily, the concerns regarding patients with hip fractures include pre-operative pain control, side effects from systemic medications, post-operative pain control, and complications including falls. It appears from the literature that the implementation of a fascia iliaca compartment block protocol could reduce the occurrence rate of all the identified concerns.

## Methods

This study was approved by the IRB at Miami Valley Hospital .

During January 2011, forty two patients were identified either as a transfer from another facility or upon presentation to the emergency department with a hip fracture. Following identification, each patient was given the option of having a continuous fascia iliaca compartment block (FICB) for pain control. All patients were included for this protocol; there were no exclusion criteria except for patient consent for participation. The anesthesiologist was contacted and consent for the procedure was obtained. The majority of the blocks were done in the emergency department with a minority being done on the orthopaedic unit for patients transferred from other hospitals. The block was performed anywhere from 1 - 4 hours after arrival to the hospital. The fascia iliaca is located anterior to the iliacus muscle within the pelvis. It is joined superior-laterally to the iliac crest and medially to fascia overlying the psoas muscle. Using ultrasound to locate the fascia layer of the psoas muscle, a needle was injected through the skin proceeding just underneath the fascia. Ultrasound permits the visualization of the needle tip piercing the fascia lata then the iliacus fascia. Local anesthetic was then injected which creates a fluid-filled space beneath the fascia. The local anesthetic travels cephalad and reaches the nerves of the lumbar plexus: the femoral, the lateral cutaneous and the obturator. This procedure is a compartment block, therefore 50 - 60 ml of local anesthetic is injected. For longer lasting analgesia, a catheter was threaded through the needle into the fascia iliaca area. A continuous infusion of local anesthetic is then joined to the catheter and maintained for several days using the On Q Pain ball. After an initial bolus of 60mL of 0.5% ropivacaine, a continuous infusion of 0.2% ropivacaine was infused at an average rate of 10mL/hr maintained until 1 - 2 days after surgery.

The block effects were monitored by the perioperative pain service. During this time, a standard protocol at our institution for patients with hip fractures was implemented including the pre-emptive receipt of Celebrex and Lyrica unless contraindicated, scheduled Tylenol, and oral and intravenous opioids as needed. This protocol also includes documentation of pain scores and medication administration including opioid consumption. The data compiled from these standardized care measures was collected as part of this study. The patients are on bedrest restrictions prior to surgery due to their fracture. The pain catheter is removed on the morning following surgery and there are no restrictions as physical therapy would ensue later that day.

## Results

**Figure 1:**
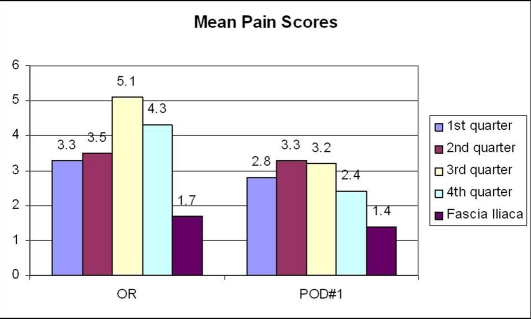
Mean pain scores post-operative.

**Figure 2:**
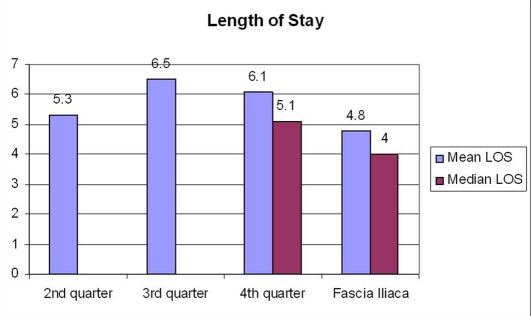
Length of stay.

The average age of this forty two person group was seventy five years (range 53 - 99 years). The group consisted of eighteen males and twenty four females. The mean pain score was averaged for each post-operative day from all pain scores recorded by the nursing staff for that day (Fig. 1). The pain score on post-operative day zero was reduced from a 2010 annual average of 4.1 to 1.7 in the pilot study group on the visual analog score. On post-operative day one, the 2010 annual average was 2.9 compared to 1.4 in the pilot study group. The length of stay was decreased from the 2010 annual average of 5.9 days to 4.8 days in the pilot study group (Fig. 2). The patients used an average of 18 mg of morphine equivalent medications during the average infusion time of 40.7 hours. There were no falls or infections noted within our pilot study group.

## Discussion

The population affected by hip fractures is a population with multiple co-morbidities. There are concerns for obtaining adequate analgesia while controlling for the amount of opioid medications used. A reduction in opioid usage during hospitalization for hip fractures reduces the risk of opioid-induced side effects: acute delirium, urinary retention, and constipation. Stevens et al completed a randomized, double blind study of total hip arthroplasty patients with a modified fascia iliaca block and found a decrease in the amount of morphine used between the control and trial groups [8].

Pain and medication use can affect the mental status of elderly patients, including the prevalence of delirium. Mouzopoulos et al found that in hip fracture patients at intermediate risk for delirium, the fascia iliaca compartment block significantly prevented the occurrence in delirium [9]. Adequate analgesia allows for increased mobility of patients and shorter hospitalizations. Dolan et al demonstrated an increased frequency of sensory loss in the medial thigh along with an increased incidence of femoral and obturator motor block with an ultrasound guided fascia iliaca block compared to the loss of resistance technique [10]. Hogh et al demonstrated an increased ability of hip flexion and a reduction of verbal pain scores in a hip fracture patient population following fascia iliaca compartment blocks placed by junior registrars [11]. Candal-Couto et al demonstrated that the fascia iliaca block allows for significant pain control pre-operatively including patients being able to tolerate a sitting position in a study of 30 patients with femoral neck fractures [12]. A reduction in length of stay is beneficial to patients in terms of reduced risk of hospital-acquired complications as well as an economic benefit for the healthcare system by reducing the length of stay.

We demonstrated the benefits of a continuous fascia iliaca compartment block placed pre-operatively when combined with a comprehensive pain protocol as measured by pain score, opioid consumption, and hospital length of stay. Regarding future studies, we have an ongoing study comparing 200 patients with continuous infusion fascia iliaca compartment blocks with 200 patients without the blocks in our hip fracture population. We plan to do a multivariate analysis of our results in addition to a cost analysis as the hip fracture population comprises a significant amount of resources.
